# Antibiotic feeding changes the bacterial community of *Chilo suppressalis* and thereby affects its pesticide tolerance

**DOI:** 10.1186/s12866-024-03421-2

**Published:** 2024-07-23

**Authors:** Xue Xia, Bing-Qian Liu, Pei-Han Yu, Zheng-Ping Yu, Ru Zhang, Guang-Hua Luo, Ji-Chao Fang

**Affiliations:** 1 Institute of Plant Protection, Jiangsu Academy of Agricultural Sciences, Nanjing, 210014 China; 2 College of Plant Protection, Nanjing Agricultural University, Nanjing, China

**Keywords:** Antibiotic, Symbiotic microorganism, *Chilo suppressalis*, Pesticide tolerance

## Abstract

**Background:**

Owing to the widespread use of chemical pesticides to control agricultural pests, pesticide tolerance has become a serious problem. In recent years, it has been found that symbiotic bacteria are related to pesticides tolerance. To investigate the potential role of microorganisms in the pesticide tolerance of *Chilo suppressalis*, this study was conducted.

**Results:**

The insect was fed with tetracycline and cefixime as the treatment group (TET and CFM, respectively), and did not add antibiotics in the control groups (CK). The 16S rDNA sequencing results showed that antibiotics reduced the diversity of *C. suppressalis* symbiotic microorganisms but did not affect their growth and development. In bioassays of the three *C. suppressalis* groups (TET, CFM, and CK), a 72 h LC_50_ fitting curve was calculated to determine whether long-term antibiotic feeding leads to a decrease in pesticide resistance. The CK group of *C. suppressalis* was used to determine the direct effect of antibiotics on pesticide tolerance using a mixture of antibiotics and pesticides. Indirect evidence suggests that antibiotics themselves did not affect the pesticide tolerance of *C. suppressalis*. The results confirmed that feeding *C. suppressalis* cefixime led to a decrease in the expression of potential tolerance genes to chlorantraniliprole.

**Conclusions:**

This study reveals the impact of antibiotic induced changes in symbiotic microorganisms on the pesticide tolerance of *C. suppressalis*, laying the foundation for studying the interaction between *C. suppressalis* and microorganisms, and also providing new ideas for the prevention and control of *C. suppressalis* and the creation of new pesticides.

**Supplementary Information:**

The online version contains supplementary material available at 10.1186/s12866-024-03421-2.

## Introduction

Many symbiotic bacteria in insects play important roles in host nutrition, development, and evolution during long-term co-evolution [1]. Microorganisms affect the establishment of pesticide tolerance in host insects [2], and there are two main types of influence. First, microorganisms in insects directly provide resistance to the host, including degrading specific pesticides and the expression of microbial detoxification metabolism genes to provide a wide range of pesticide resistance to the host. For example, *Riptortus pedestris* rapidly develops resistance to organophosphate pesticides through *Burkholderia*, which is obtained from the soil and colonizes the gut and can spread horizontally [3]. Second, host pesticide toleranceis improved mainly through the upregulation of immune or detoxification metabolism-related pathways [4]. For example, infection with secondary symbiotic *Hamiltonella defensesa* may increase detoxification-related enzymatic activity in the wheat aphid *Sitobion miscanthi*, thereby reducing pesticide sensitivity in the host [5].

Rice is one of the most important food crops and supports nearly half of the world’s population. *Chilo suppressalis* is an important rice pest that affects almost all rice-growing areas in China and many other rice-growing countries and regions [6]. In China, chemical control is the main method to suppress *C. suppressalis*, although biological control is also used. Heavy insecticide use has resulted in the development of tolerance to many conventional insecticides, including organochlorine, organophosphorus, and nereistoxin [7, 8]. Methoxyfenazide and chlorantraniliprole are commonly used to control chemical pesticides on *C. suppressalis* in China. The endosymbiotic bacterium *Wolbachia* in *C. suppressalis* reduces its sensitivity to abamectin and fipronil and improves its pesticide tolerance [9]. The diversity of gut microbiota in Bt-resistant populations of *C. suppressalis* (BJ1Ab-R and FZ1Ca-R) is also significantly higher than that in Bt-sensitive populations (BJ-S and FZ-S) [10]. It is not clear whether other symbiotic microorganisms affect the pesticide resistance of *C. suppressalis*.

To further determine the effects of symbiotic microorganisms in *C. suppressalis*, especially intestinal bacteria, on growth, development, and pesticide tolerance, this study used lab-reared pesticide-sensitive *C. suppressalis* as research objects, fed an artificial diet with different antibiotics after multiple generations, and then obtained populations of *C. suppressalis* with different bacterial communities. The 16S rDNA sequencing was used to identify the differences in symbiotic bacterial communities and determine the effects of different antibiotics on these microbial communities. Simultaneously, biological indices, including larval developmental period, pupal stage, pupal weight, and adult life span, were recorded to determine the effects of microbial population changes caused by antibiotics on the growth and development of *C. suppressalis*. The pesticides chlorantraniliprole and methoxyfenozide were used to determine and calculate the 72 h LC_50_ of *C. suppressalis* fed with different antibiotics. The results revealed the differences in the pesticide tolerance of *C. suppressalis* fed different antibiotics. Simultaneously, a population of *C. suppressalis* without antibiotics was used as the study target, and the direct effect of antibiotics on the tolerance of *C. suppressalis* was determined by the mixed use of pesticides and antibiotics. Thus, the relationship between changes in the intestinal microbial population and the pesticide tolerance of *C. suppressalis* fed different antibiotics was indirectly determined. To determine the effect of antibiotic feeding on the genes involved in chlorantraniliprole detoxification metabolism in *C. suppressalis*, quantitative reverse transcription PCR (RT-qPCR) was used to quantify gene expression.

## Materials and methods

### Artificial breeding of *C. suppressalis*

The *C. suppressalis* population used in this study was collected from rice paddies around the Jiangxi Academy of Agricultural Sciences in Jiangxi, China (Longitude: 115.941823 Latitude: 28.558001), and raised in the laboratory for more than 10 years (at least 100 generations). The collected insects were bred in laboratory at 28 ± 1℃ with a photoperiod of 16 h light:8 h dark (L: D), and a relative humidity of 70 − 80%. The method of artificial feeding was referred to the patents [11]. In order to obtain *C. suppressalis* group with different microbial communities, *C. suppressalis* were treated with 1 g/L concentration of tetracycline and 0.05 g/L concentration of cefixime in artificial feed, which were referred to as TET and CFM, respectively. The group of *C. suppressalis* without antibiotics in artificial feed were referred to as control check (CK). The additive concentrations of tetracycline and cefixime were selected according to existing research [12] and laboratory experience [11]. All three groups of *C. suppressalis* were raised in the laboratory for more than 5 generations for follow-up experiments. Each group ensures that there were about 2,000 larvae per generation and at least 800 adults survive for generational breeding.

### 16 S rDNA amplicon sequencing

To perform 16S rDNA sequencing, the 4th instar larvae of three different groups of *C. suppressalis*, including TET, CFM, and CK, were collected and washed with 75% alcohol on the body surface. They were then frozen and stored in liquid nitrogen. Each group had 5 biological samples, and each biological sample had three 4th instar larvae of *C. suppressalis*. According to manufacturer’s protocols., HiPure Stool DNA Kits (Magen, China) were used to extract total genomic DNA. Generate an amplified library using the two-step PCR method recommended by Illumina [13]. Specifically, PCR amplification of the 16S rDNA gene in bacteria involves universal primers 341 F (5’-CCTACGGGNGGCWGCAG-3’) and 806R (5’-GGACTACHVGGGTATCTAAT-3’) with barcode [14]. Q5^®^ Action Buffer Pack and Q5^®^ High GC Enhancer (NEB, USA) were used for PCR amplification. The PCR products were evaluated with 2% agarose gels and purified using AMPure XP Beads (Beckman, USA) and quantified using Qubit 3.0 (Invitrogen, USA). After the PCR product purification, the sequencing libraries were generated using Illumina DNA Prep Kit (Illumina, USA). The library quality was assessed with the ABI StepOnePlus Real Time PCR System (Life Technologies, USA). Purified amplicons were pooled in equimolar and paired-end sequenced (PE250) on an Illumina Novaseq 6000 platform according to the standard protocols [15].

### Data quality control, denoising, clustering, and annotation

The raw reads obtained from sequencing were subjected to data filtering, quality control, clustering, and other processes by using DADA2 R package. Specifically, DADA2 removed sequencing primers at both ends and low-quality reads containing unknown nucleotides (N bases) from raw reads and output non-redundant reads and corresponding abundance information. Meanwhile, DADA2 used machine learning to construct the error model for reads denoising, by alternately estimating the error rate and learning the error model from the reference sample sequence until the learning model conversions to the true error rate. Then, a dereplicated list of unique sequences and their abundances were output, as well as the consensus positional quality scores for each unique sequence by taking the average (mean) of the positional qualities of the component reads. These consensus scores were used by the error model. The denoised reads were concatenated into Tags, and the UCHIME algorithm was used to identify and delete the chimeric sequence [16], obtaining the Tag sequence and abundance information for subsequent analysis, namely the amplicon sequence variants (ASVs) sequence and ASV abundance information. The representative ASV sequences were classified into organisms by a naive Bayesian model using RDP classifier [17] (version 2.2) based on SILVA database [18] (version 138.1) with the confidence threshold value of 0.8.

### Analysis of species composition and microbial diversity

The stacked bar plot of the community composition was visualized in R project ggplot2 package [19] (version 2.2.1). Between groups Venn analysis was performed in R project VennDiagram package [20] (version1.6.16) and upset plot was performed in R project UpSetR package [21] (version 1.3.3) to identify unique and common species or ASVs. Heatmap of different taxonomic categories abundance was plotted using pheatmap package (version 1.0.12) in R project.

Chao1, Shannon, Simpson, Pielou’s evenness index were calculated in QIIME [22] (version 1.9.1). PD-whole tree index was calculated in picante [23] (version 1.8.2). Alpha index comparison among groups was computed by Tukey’s HSD test in R project Vegan package (version 2.5.3) and plotted in GraphPad prism 8 (GraphPad Software, USA).

Sequence alignment was performed using Muscle [24] (version 3.8.31) and phylogenetic tree was constructed using FastTree [25] (version 2.1), then weighted unifrac distance matrix were generated by GuniFrac package [26] (version 1.0) in R project. Principal coordinate analysis (PCoA) of weighted unifrac distances were generated in R project Vegan package (version 2.5.3) and plotted in R project ggplot2 package (version 2.2.1). Statistical analysis Tukey’s HSD test was calculated in R project Vegan package (version 2.5.3).

### Development indicators of the *C. suppressalis* fed with different antibiotics

The 1-day-old larvae of the newly hatched *C. suppressalis* were raised in a single head in a glass finger tubes (Height 108 mm and bottom diameter 27.5 mm) with self-made cotton stoppers, and artificial feed with different antibiotics was cut into approximately 1 cm^3^. The feed was changed every three days. Record the status of the *C. suppressalis* every day, using the head shell as the main indicator of larval age changes, and record the different developmental stages of the *C. suppressalis* larvae. The larval stage is terminated by the occurrence of pre-pupation (refusal to feed, shortening and shrinking of the larval body), followed by recording the pupal stage, distinguishing between male and female, and measuring the weight of a single head pupa. After the emergence of virgin male and female insects, the adult lifespan was recorded using 10% sucrose water as feed. At least 35 biological replicates per group.

### Bioassay

Different concentrations of pesticides were used to immerse artificial feed with different antibiotics and then raised the *C. suppressalis*, recording the mortality rate at 72 h, and determining the LC_50_. The method was improved by referring to soaking rice leaves [27]. Specifically, artificial feed cut into 1 cm^3^ was soaked 60 s in an aqueous solution with different concentrations of pesticides (Includes 6 concentrations, 2 mg/L, 1 mg/L, 0.5 mg/L, 0.25 mg/L, 0.125 mg/L and 0.0625 mg/L), and then air dry in a clean workbench for 5 min. Subsequently, the dried artificial feed was put in a glass finger tube. 10 newly hatched 2-day-old larvae of *C. suppressalis* were put on 1 piece of 1 cm^3^ feed in each finger-shaped tube as a biological replicate. Each concentration was treated with at least 3 biological replicates. After 72 h of feeding, the number of larval deaths was recorded, and the mortality rate was calculated. The 72 h LC_50_ fitting curve was drawing and 72 h LC_50_ was calculated by Graphpad prism 8.

To determine the direct impact of antibiotics on the pesticide tolerance of the *C. suppressalis*, a mixture of 1 mg/L pesticides and different antibiotic solutions to treat the *C. suppressalis* raised on CK group as treatment group, and the *C. suppressalis* raised on artificial feed treated with 1 mg/L pesticides alone as the blank control group. As mentioned earlier, the mortality rate was recorded after 72 h of feeding in a glass finger tube. The treatment group and control group both contained 10 biological replicates.

The following insecticides used in this assay were technical grade: chlorantraniliprole (95.3%) and methoxyfenozide (97%). These two insecticides were provided by Syngenta (China) Investment Co., Ltd and Jinan Tianbang Chemical Co., Ltd. A stock solution containing 10 g/L effective components was prepared by dissolving this pesticide technical grade in dimethylformamide.

### RNA extraction and RT-qPCR

In order to determine the effect of antibiotic feeding on the potential detoxification metabolism genes of chlorantraniliprole in *C. suppressalis*, based on previous studies, genes potentially involved in chlorantraniliprole metabolism were selected from *C. suppressalis* for RT-qPCR in this study [28]. The primer sequences are shown in the table below (Table S1). The third instar larvae were treated with method as above and RNA was extracted. The three control groups without chlorantraniliprole treatment were called CK, TET and CFM, respectively, which corresponded to the groups fed with different antibiotics and without antibiotics. The other three groups that were treated with chlorantraniliprole were called CK_T, TET_T and CFM_T. The specific methods were as follows: 1 mg/L chlorantraniliprole aqueous solution was used to treat CK, CFM and TET groups for 24 h, liquid nitrogen quick-freezing was used as the treatment group, and CK, CFM and TET groups without pesticide treatment were used as the control group with liquid nitrogen quick-freezing. There were 6 groups with at least 4 biological replications in each group. Each biological replicate had 3–4 third instar larvae, a total of 24 biological replicates.

According to the manufacturer’s instructions, the EASYspin Plus rapid tissue/Cell RNA Extraction kit (Aidlab, Beijing) was used to extract the above 24 biological replicates. The RNA quantity was measured with an Eppendorf BioPhotometer D30 (Eppendorf, Germany), and RNA quality was measured with 1% agarose gel electrophoresis. First-strand cDNA synthesis was conducted with 1000 ng of total RNA from each sample using the PrimeScript™ RT reagent Kit with gDNA Eraser (Perfect Real Time) according to manufacturer’s instructions (Takara, Japan). cDNAs were then diluted 1: 10 in nuclease-free water and stored at -20 °C. Amplification was carried out using the LightCycler^®^ 480 Instrument II (Roche, Switzerland) as follows: 3 min at 95 °C; 40 cycles of 5 s at 95℃, and 34 s at 60℃. The dissolution curve was formed from 55–98℃. Each sample contained 20 µL total reaction volume, which contained 5 pmol of each primer, 10 µL Hieff UNICON^®^ qPCR SYBR Green Master Mix (Yeason, China), and 2 µL diluted cDNA. Each sample had two technical repeats. The relative expression levels were calculated using the 2^−ΔΔCt^ method [29].

### Statistical analysis

One-way ANOVA (differences among multiple groups) and Student’s t-test (differences between two groups) were used to analyze the data. Post hoc multiple comparisons for multiple groups were performed Tukey-HSD test in one-way ANOVA, and differences were regarded as statistically significant as *P* < 0.05. All statistical analyses were performed using the Statistical Package for the Social Sciences v25.0 software (IBM, USA).

## Results

### Composition of the bacterial community in *C. suppressalis* treated with different antibiotics

**Fig. 1:**
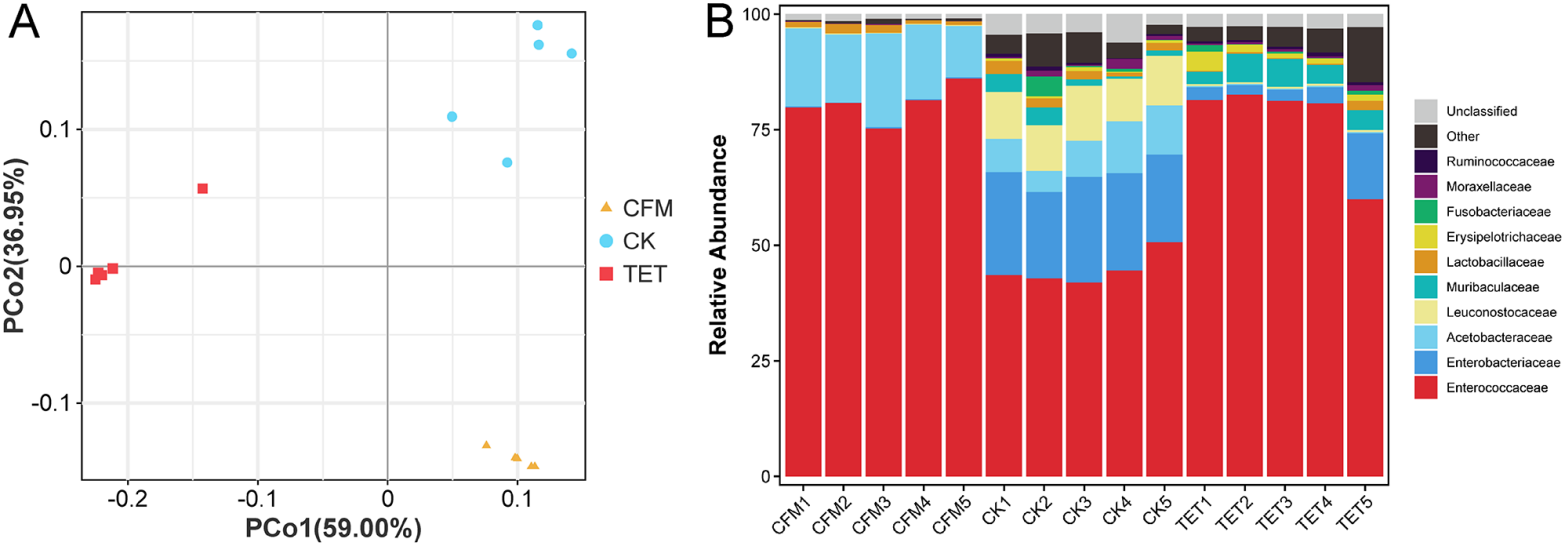
16S rDNA sequencing principal co-ordinates analysis (PCoA) and Species distribution histogram at family level. (**A**) PCoA analysis diagram of different samples of *Chilo suppressalis* Each point in the diagram represents a sample, and the closer the points on the plane are, the more similar the microbial community structure of the sample is. (**B**) Species stacking map of different samples at family levels Only species with an average abundance of top10 are displayed in all samples

PCoA results showed that the different groups of *C. suppressalis* treated with different antibiotics exhibited clear aggregation. Independent biological replicates were generally consistent but were more variable among samples in the CK group (Fig. 1A). Different taxonomic levels annotated by all ASVs in the different samples showed that the proportion annotated to genera was the highest, and the proportion annotated to families and lower taxonomic levels exceeded 95% (Fig S1, Additional file 1). Therefore, the species distribution stack map only displays results at the family level. The three most abundant families were Enterococcaceae, Enterobacteriaceae, and Acetobacteraceae. Enterococcaceae had the highest proportion among all three groups, with CFM (80.66%), TET (77.18%), and CK (44.70%) ranking from highest to lowest. The second-highest proportion of Enterobacteriaceae was extremely low in the CFM group (0.23%), followed by the TET (5.02%) and CK (20.78%) groups. The third highest proportion of Acetobacteraceae was extremely low in the TET (0.26%), CFM (15.83%), and CK (8.28%) treatments (Fig. 1B).**3.2 Different antibiotics have different effects on the richness and evenness of the bacterial population in*C. suppressalis***.

**Fig. 2:**
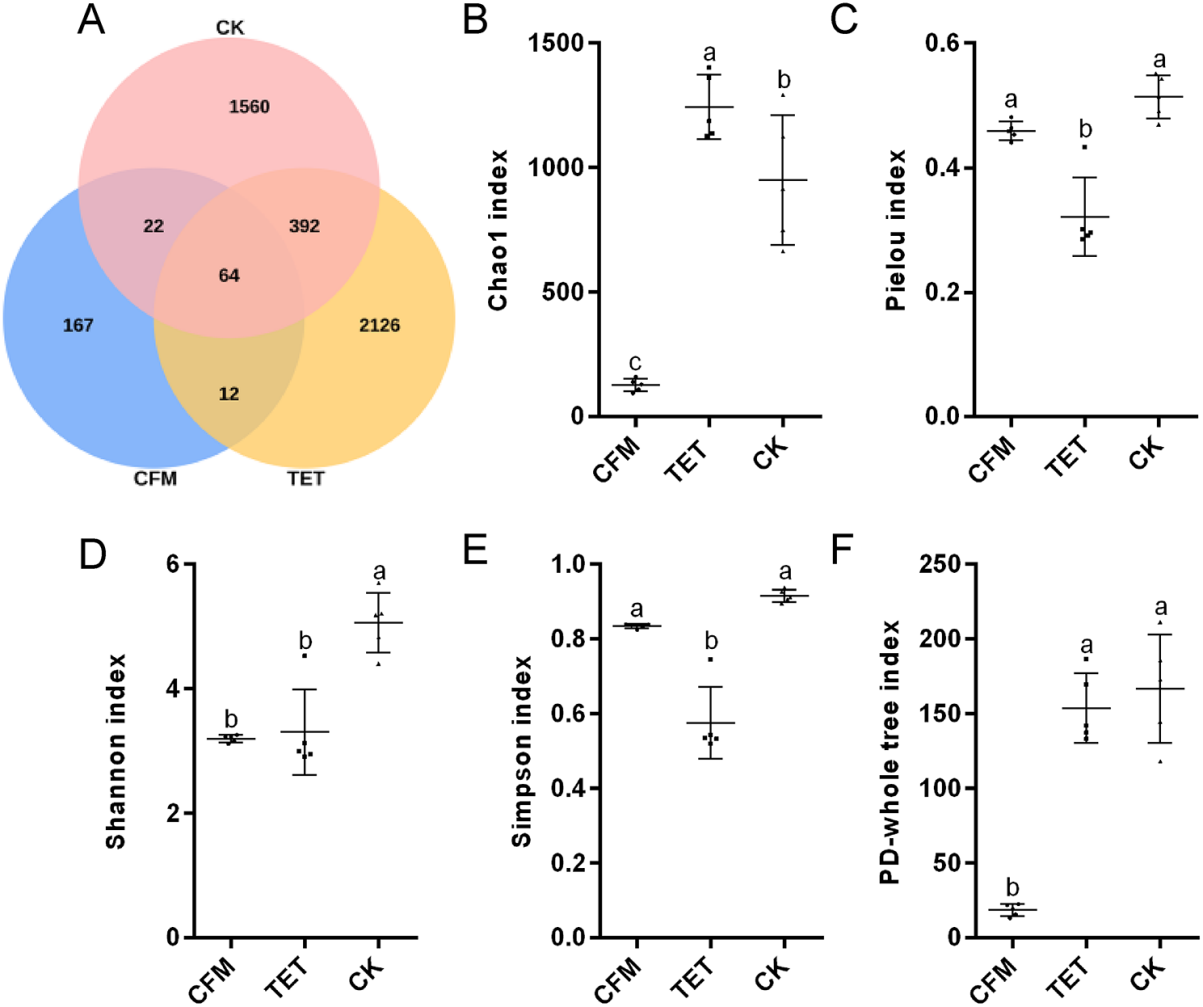
Diversity analysis of 16S rDNA sequencing results. **A**. ASVs Venn plot of symbiotic microorganisms in *C. suppressalis* fed with different antibiotics. **B**, **C**, **D**, **E** and **F**. Alpha diversity index of symbiotic microorganisms in *C. suppressalis* fed with different antibiotics. Commonly used α diversity indices such as Chao1, Pielou, Shannon, Simpson and PD-whole tree and their correlation analysis results.The data is represented by mean ± SD. Lowercase letters indicate significant differences (Tukey HSD test, *P* < 0.05); NS indicates no significant difference

The addition of cefixime to the artificial feed of *C. suppressalis* significantly reduced bacterial population richness compared to the addition of tetracycline (TET) and the control (CK). The addition of cefixime (CFM) to artificial feed significantly reduced the number of ASVs in *C. suppressalis* compared to those fed with tetracycline and artificial feed without antibiotics (Fig. 2A). The ASVs numbers in the three groups, from highest to lowest, were in the order of TET (2594) > CK (2038) > CFM (265). The Chao1 value, based on the alpha diversity index also indicated the same result, with the species richness of the samples in the three treatments in the order, TET > CK > CFM. There was a significant difference between any two group (Fig. 2B). Therefore, we determined that cefixime significantly reduced the bacterial population richness in *C. suppressalis*, whereas tetracycline slightly increased the bacterial population richness in *C. suppressalis*.

The addition of tetracycline to the artificial feed of *C. suppressalis* significantly reduced bacterial population evenness compared to the addition of cefixime and the absence of antibiotics. Pielou’s evenness index indicated this result (Fig. 2C), and the bacterial population evenness in the three groups was in the order of CK > CFM > TET from high to low.

### TET and CFM significantly reduce the microbial community diversity of *C. suppressalis*

The addition of antibiotics to the artificial feed decreased the microbial population diversity of *C. suppressalis*. The Simpson, Shannon, and PD whole tree indices, based on the alpha diversity index, showed that the CK group had the highest microbial community diversity (Fig. 2D, E, F). The Simpson index indicated that the microbial community diversity of the CFM group was significantly greater than that of the TET group. The PD-tree index showed that the lineage diversity of the TET group was significantly greater than that of the CFM group, which may be because the Simpson index is greatly affected by evenness. Combined with the above results of richness and evenness of the bacterial population in *C. suppressalis* (Fig. 2B, C), it can be concluded that the TET contained more ASVs (Fig. 2A), that is, a higher species richness. However, the sequence number of different ASVs varied greatly, and multiple ASVs contained fewer sequence numbers (Additional file 1), that is, the evenness was low. The PD-whole tree index is based on calculating the sum of the total phylogenetic branch lengths to assess the degree of diversity. As the number of ASVs in the TET group was significantly higher than that in the CFM group, the phylogenetic diversity in the TET group was significantly higher than that in the CFM group. Overall, the addition of antibiotics to the artificial feed reduced the microbial diversity of *C. suppressalis*.

### Antibiotics do not affect the growth and development of *C. suppressalis* on artificial feed

**Fig. 3:**
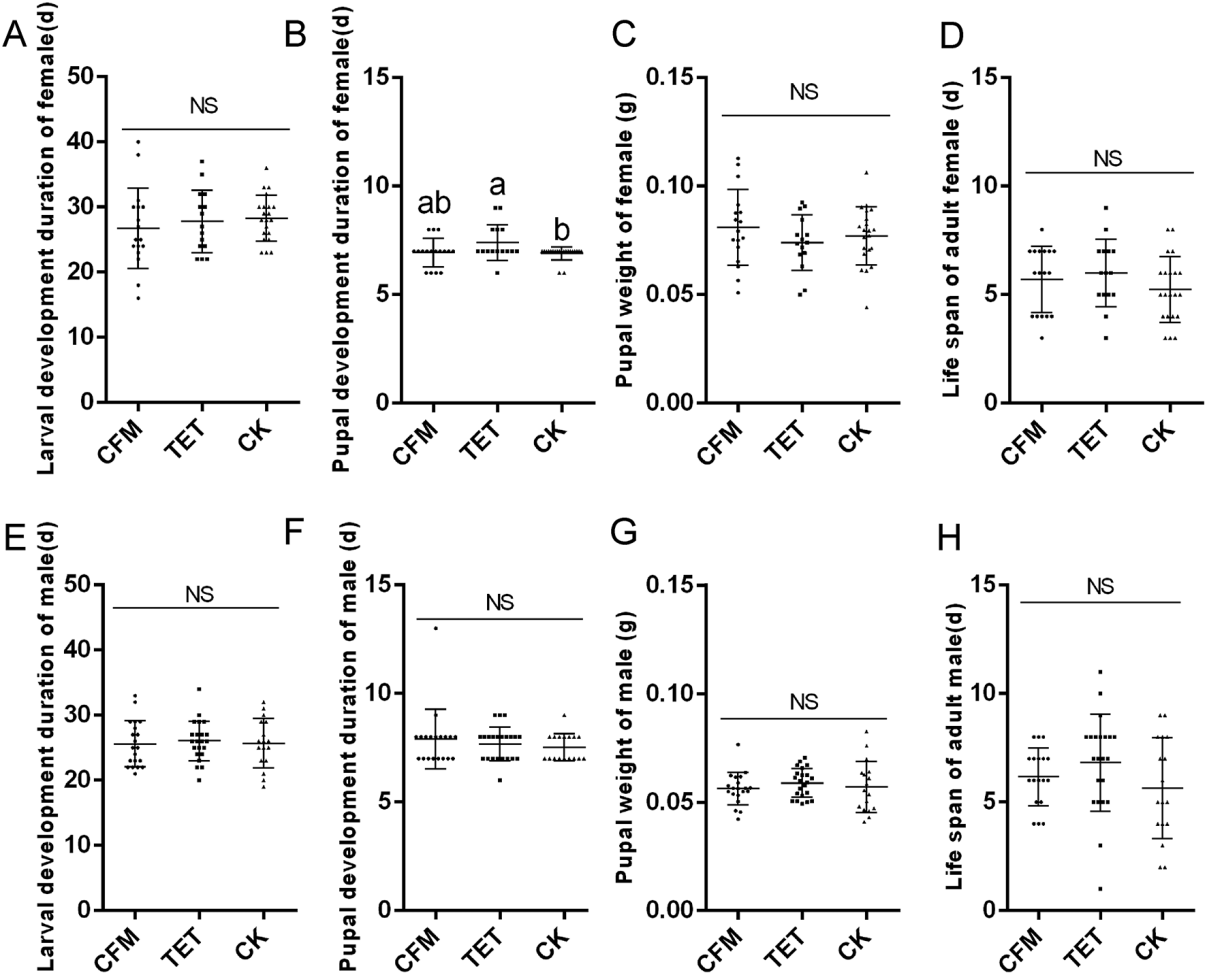
Comparison of growth and development indicators between groups of *Chilo suppressalis* fed with different antibiotic. Female larva development duration (**A**), female pupal development duration (**B**), female adult life span (**C**), and female pupal weight (**D**) in *C. suppressalis* population fed with different antibiotic. Male larva development duration (**E**), male pupal development duration (**F**), male adult life span (**G**), and male pupal weight (**H**) in *C. suppressalis* population fed with different antibiotics.The X-axis represents the population grouping of *C. suppressalis* fed with different antibiotics, while the Y-axis represents different biological indicators. The data is represented by mean ± SD. Lowercase letters indicate significant differences (Tukey HSD test, *P* < 0.05); NS indicates no significant difference

Artificial feed supplemented with different antibiotics was used to feed the *C. suppressalis* in glass finger tubes, and the results showed that among different groups, except for the pupal development period of the females, development of the TET group was significantly longer than that of the CK group (ANOVA, *F* = 3.378, df = 2,50, *P* < 0.05). There were no significant differences in other indicators, such as larval development period, male pupal development period, pupal weight, and adult lifespan (Fig. 3). These results revealed that the addition of antibiotics to artificial feed had no significant effect on the growth and development of the *C. suppressalis* under an artificial diet.

### Long-term feeding of antibiotics leads to decreased tolerance of *C. suppressalis* to chlorantraniliprole and methoxyfenozide

**Fig. 4:**
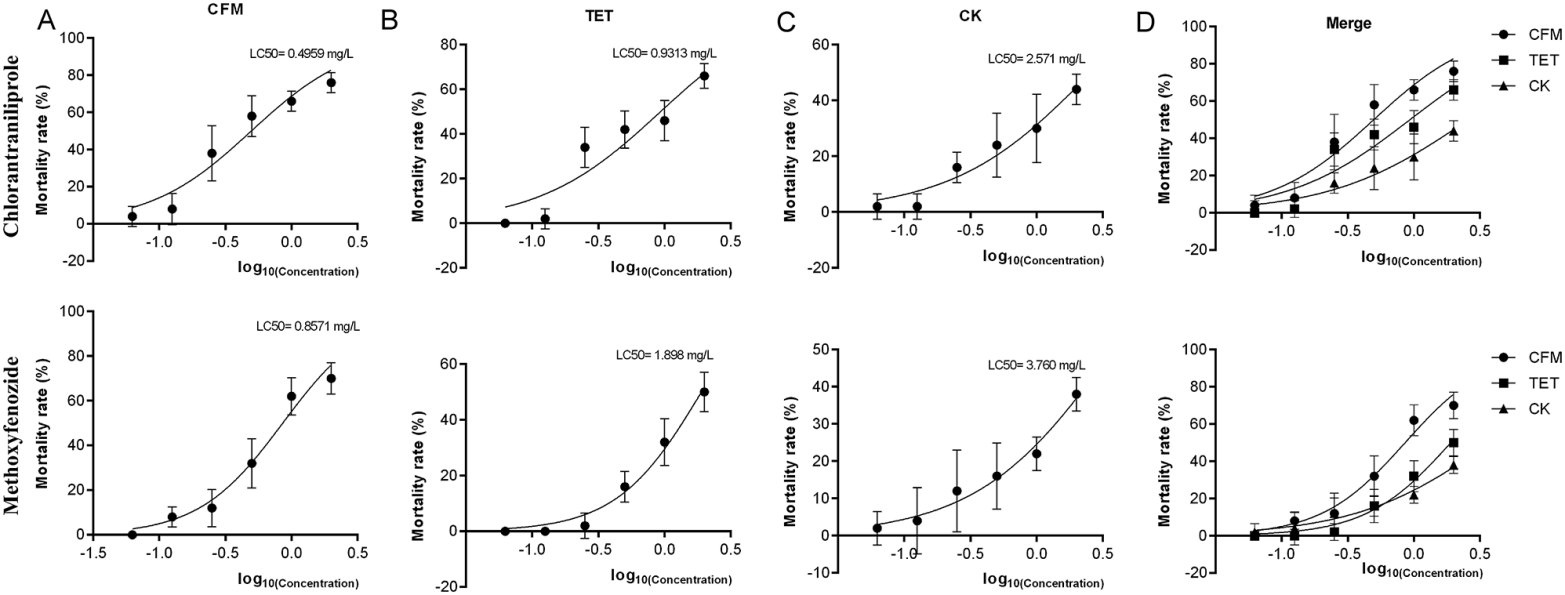
LC_50_ fitting curve for chlorantraniliprole and methoxyfenozide in *C. suppressalis* (**A**). LC_50_ fitting curve for chlorantraniliprole and methoxyfenozide in CFM group (**B**). LC_50_ fitting curve for chlorantraniliprole and methoxyfenozide in TET group (**C**). LC_50_ fitting curve of chlorantraniliprole and methoxyfenozide in CK group (**D**). Aggregation of LC_50_ fitting curves of chlorantraniliprole and methoxyfenozide for three groups of *C. suppressalis.* The X-axis represents log_10_ (pesticide concentration), while the Y-axis represents mortality rate. The data is represented by mean ± SD. Each data point contains 5 biological replicates. The top right corner indicates the best hit value of LC_50_ calculated for each curve

Compared to the CK group, the population of *C. suppressalis* fed with artificial feed.

supplemented with different antibiotics showed a decrease in the 72 h LC_50_ of both chlorantraniliprole and methoxyfenozide (Fig. 4). *C. suppressalis* fed with 0.05 g/L CFM was the most sensitive to both pesticides, whereas *C. suppressalis* fed with artificial feed without antibiotics was the least sensitive to both pesticides. The 72 h LC_50_ of chlorantraniliprole in the CFM group was 0.4959 mg/L, whereas the 72 h LC_50_ of methoxyfenozide in the CFM group was 0.8571 mg/L. (Fig. 4A). The 72 h LC_50_ of chlorantraniliprole in the TET group was 0.9313 mg/L, whereas the 72 h LC_50_ of methoxyfenozide in the TET group was 1.898 mg/L (Fig. 4B). The 72 h LC_50_ of chlorantraniliprole in the CK group was 2.571 mg/L, whereas the 72 h LC_50_ of methoxyfenozide in the CK group was 3.760 mg/L (Fig. 4C). According to the 72 h LC_50_ fitting curve, the *C. suppressalis* fed with tetracycline showed lower mortality rates than the CK group at low concentrations of chlorantraniliprole (Fig. 4D). The analysis of variance was performed on the mortality of C. suppressalis after treating with different concentrations of chlorantraniliprole and methoxyfenozide. The results showed that there was no significant difference (ANOVA, *P* > 0.05) among the CK, TET, and CFM groups after feeding with low concentrations of chlorantraniliprole (0.0625 mg/L and 0.125 mg/L) and methoxyfenozide (0.0625 mg/L, 0.125 mg/L and 0.25 mg/L) (Fig S2). When the concentration increased, different pesticide treatments showed the same trend, with the CK group having the lowest mortality rate, followed by the TET group, and the CFM group having the highest mortality rate. There was a significant difference (ANOVA, *P* > 0.05) in mortality rate between the three groups in the treatment with 2 mg/L methoxyfenozide (Fig S2A), and this phenomenon occurred at lower concentrations (> 1 mg/L) in chlorantraniliprole (Fig S2B).

### Antibiotics do not directly affect the tolerance of *C. suppressalis* to chlorantraniliprole and methoxyfenozide

**Fig. 5:**
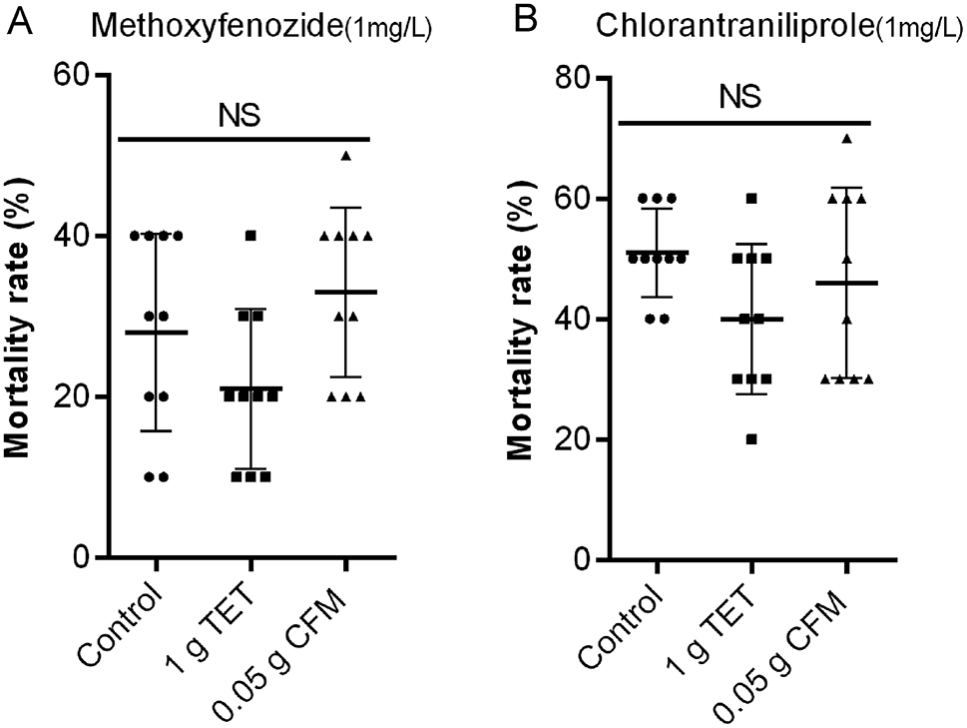
Effects of different antibiotic treatments on pesticide tolerance of CK group *C. suppressalis* (A) Effects of different antibiotic mixtures of methoxyfenozide on pesticide tolerance of CK group *C. suppressalis* (B) Effects of different antibiotics mixtures of chlorantraniliprole on pesticide tolerance of CK group *(C) suppressalis*. The X-axis represents different treatment methods for CK group, control represents 1 mg/L pesticide treatment for CK group, and 1 mg TET means that the CK group was treated with 1 mg/L tetracycline and 1 mg/L pesticide, and 0.05 g CFM means that the CK group was treated with 0.05 mg/L cefixime and 1 mg/L pesticide. The Y-axis shows the mortality rate of *C. suppressalis*. The data is represented by mean ± SD. NS indicates no significant difference (Tukey HSD test, *P* < 0.05)

To determine the direct impact of antibiotics on the pesticide tolerance of *C. suppressalis*, the CK group was treated with a mixture of antibiotics and pesticides. The results showed that the mixed use of 1 g/L tetracycline and 0.05 g/L cefixime with chlorantraniliprole and methoxyfenozide did not lead to an increase in the mortality rate of *C. suppressalis* in the CK group (Fig. 5). The average mortality rates of CK treated with 1 mg/L chlorantraniliprole and 1 mg/L methoxyfenozide were 51% and 28%, respectively. The CK group treated with antibiotic-mixed pesticides, especially tetracycline mixed pesticides, even reduced the lethal effects of chlorantraniliprole and methoxyfenozide on *C. suppressalis*, with an average mortality rate of 40% and 21%, respectively. The average mortality rates in the CK group treated with cefixime mixed with chlorantraniliprole and methoxyfenozide were 46% and 33%, respectively. These results revealed that antibiotics did not lead to a decrease in the tolerance of *C. suppressalis* and may even increase its tolerance.

### Long-term feeding of *C. suppressalis* with antibiotics downregulates detoxification metabolism-related genes

**Fig. 6:**
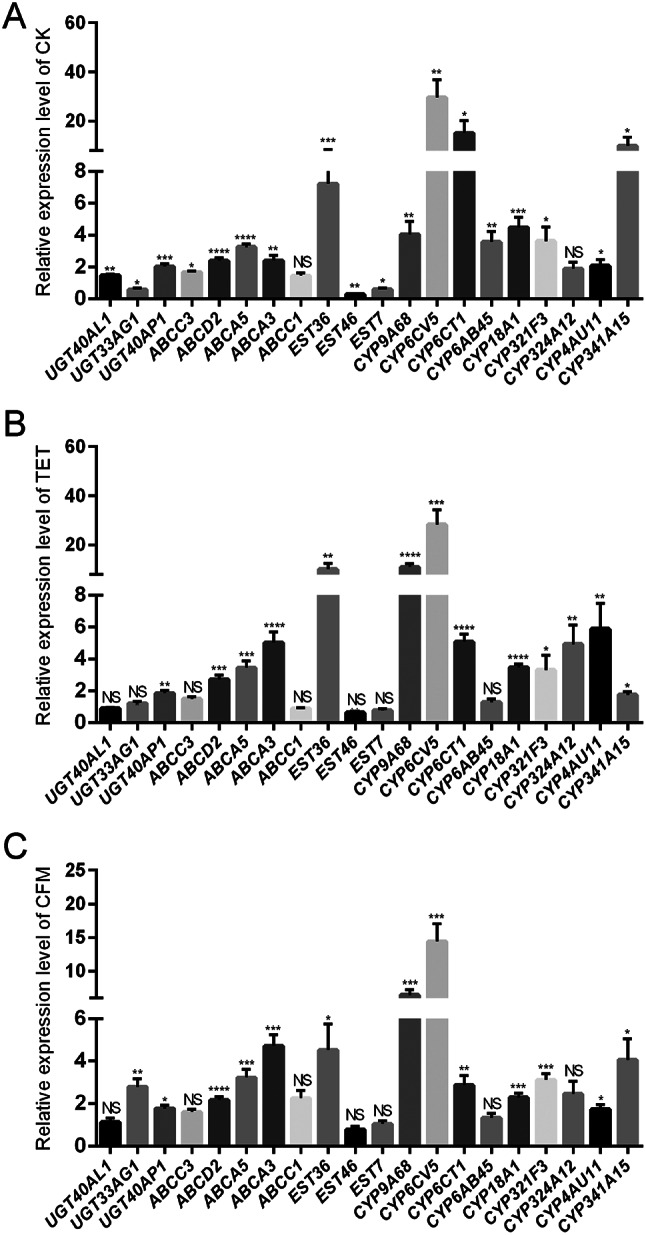
Expression level of detoxification metabolism genes in different groups of *Chilo suppressalis* after chlorantraniliprole treatment (A) Differences in the expression of detoxification metabolism genes among CK group after chlorantraniliprole treatment (B) Differences in the expression of detoxification metabolism genes among TET group after chlorantraniliprole treatment (C) Differences in the expression of detoxification metabolism genes among CFM group after chlorantraniliprole treatment. X-axis represents different genes in *C. suppressalis.* The Y-axis represents the relative expression level of genes calculated by the 2^−ΔΔCt^ method. The data is represented by mean ± SD. Each data column contains 4 biological replicates. There was a significant difference (T-test) at *P* < 0.05 (*), *P* < 0.01 (* *), *P* < 0.001 (* * *), and *P* < 0.0001 (* * *.*); NS indicates no significant difference

Using CK, TET, and CFM as the control groups, the three groups of *C. suppressalis* were treated with chlorantraniliprole. RT-qPCR results showed that the detoxification metabolism-related genes upregulated by chlorantraniliprole in all three pairs of *C. suppressalis* were *UGT40AP1*, *ABCD2*, *ABCA5*, *ABCA3*, *EST36*, *CYP9A68*, *CYP6CV5*, *CYP6CT1*, *CYP18A1*, *CYP321F3*, *CYP4AU11*, and *CYP341A15*. These results indicate that these genes are involved in the detoxification and metabolism of chlorantraniliprole. The genes that were down-regulated or not significantly differentially expressed in all three pairs of *C. suppressalis* were *ABCC1*, *EST46*, and *EST7* (Fig. 6), these genes may not involve in the detoxification and metabolism of chlorantraniliprole. At least the expression levels were not upregulated by chlorantraniliprole.

**Fig. 7:**
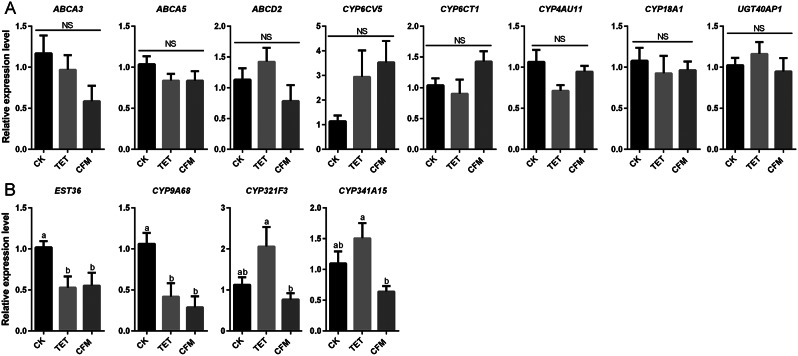
Expression level of detoxification metabolism genes of chlorantraniliprole in *C. suppressalis* caused by different antibiotic feeding. (A) Detoxification metabolism genes with no significant difference in expression among the CK, CFM, and TET groups. (B) Detoxification metabolism genes with significant difference in expression among the CK, CFM, and TET groups. The X-axis represents different groups of *(C) suppressalis*, and the Y-axis represents the relative gene expression level calculated using the 2^−ΔΔCt^ with the CK group as the control. The data is represented by mean ± SD. Each data column contains 4 biological replicates. Lowercase letters indicate significant differences (Tukey HSD test, *P* < 0.05); NS indicates no significant difference

Further analysis was conducted on the changes in the expression of 12 genes (Fig. 6) that were identified to be involved in the detoxification metabolism of *C. suppressalis*, using the CK group as the control and the TET and CFM groups as the treatment groups. The results showed that there was no significant difference in the expression of 8 of 12 genes among the CK, CFM, and TET groups, including *UGTAP1*, *ABCD2*, *ABCA5*, *ABCA3*, *CYP6CV5*, *CYP6CT1*, *CYP18A1* and *CYP4AU11* (Fig. 7A). Four differentially expressed genes, including *EST36*, *CYP9A68*, *CYP321F3* and *CYP341A15*, showed the lowest relative expression levels in the CFM group. Both *EST36* and *CYP9A68* showed the highest relative expression levels in the CK group, and they were significantly different compared to both the TET and CFM groups. *CYP321F3* and *CYP341A15* had the highest expression levels in the TET group, but there was no significant difference in their relative expression levels compared with the CK group (Fig. 7B).

**Fig. 8:**
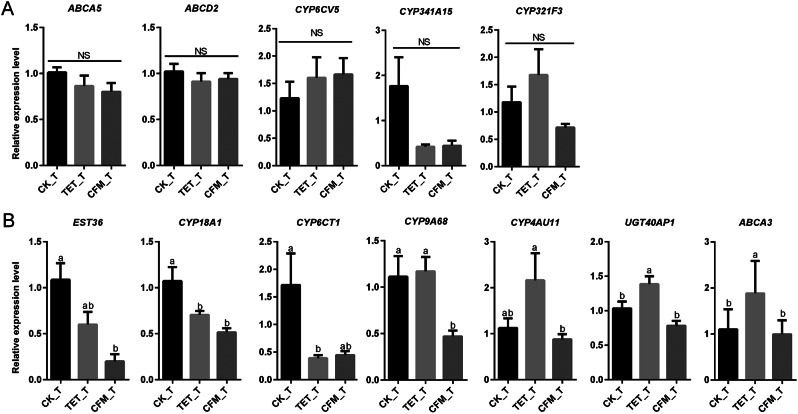
Expression level of detoxification metabolism genes between different groups of *C. suppressalis* after chlorantraniliprole treatment. (A) Detoxification metabolism genes with no significant difference in expression among the CK_T, CFM_T, and TET_T groups. (B) Detoxification metabolism genes with significant difference in expression among the CK_T, CFM_T, and TET_T groups. The X-axis represents different groups of *(C) suppressalis*, and the Y-axis represents the relative gene expression level calculated using the 2^−ΔΔCt^ with the CK_T group as the control. The data is represented by mean ± SD. Each data column contains 4 biological replicates. Lowercase letters indicate significant differences (Tukey HSD test, *P* < 0.05); NS indicates no significant difference

The CK group of *C. suppressalis* treated with chlorantraniliprole (CK_T) was used as the control group, and the TET and CFM groups of *C. suppressalis* treated with chlorantraniliprole were used as the treatment groups (TET_T and CFM_T). The expression changes of 12 genes (Fig. 6) identified as being involved in the detoxification metabolism of *C. suppressalis* were analyzed in different groups treated with chlorantraniliprole. The results showed that the relative expression levels of *ABCA5*, *ABCD2*, *CYP6CV5*, *CYP321F3 and CYP341A15* were not significantly different between different groups of *C. suppressalis* treated with chlorantraniliprole. The seven differentially expressed genes were *EST36*, *CYP18A1*, *CYP9A68*, *CYP6CT1*, *CYP4AU11*, *UGT40AP1*, and *ABCA3* (Fig. 8A). These differentially expressed genes also showed the lowest relative expression levels in the CFM group (Fig. 8B), with varying levels of relative expression in the CK and TET groups.

## Discussion

Long-term addition of tetracycline to the artificial feed led to an increase in the number of symbiotic ASVs in *C. suppressalis*, which may be related to the accumulation of tetracycline tolerance in environmental microorganisms and the inhibition of Acetobacteraceae by tetracycline. Resistance of environmental bacteria to tetracycline has always been an important research topic [30–32]. In 2011, a broad-spectrum survey was conducted in selected European countries, and the tetracycline resistance rates of *Escherichia coli* and *Klebsiella.spp* producing broad-spectrum β-lactamase were 66.9% and 44.9%, respectively [33]. In 2015, the global tetracycline resistance rates of methicillin-resistant *Staphylococcus aureus* (MRSA) and *Streptococcus pneumoniae* were 8.7% and 24.3%, respectively [34]. In this study, it was found that, compared with the CK group without antibiotics, although the microbial diversity and evenness decreased in the TET group, the microbial richness increased; that is, the number of ASVs increased (Fig. 2A). These results suggest that the bactericidal effect of tetracycline on the symbiotic microbial community of *C. suppressalis* was not ideal, or even very low, which may mean that there is widespread tetracycline resistance in environmental microorganisms. Moreover, even if tetracycline had no killing effect on the symbiotic microorganisms of *C. suppressalis*, it should not cause an increase in the number of ASVs. Therefore, it is speculated that this phenomenon is also related to the significant decrease in the proportion of Acetobacteraceae in *C. suppressalis* caused by tetracycline. Acetobacteraceae includes a wide range of Gram-negative obligate aerobic bacteria. They occur primarily in sugary, acidic, and alcoholic environments, and have been extensively studied because of their positive, neutral, or harmful roles in food and beverage manufacturing [35, 36]. Acetobacteraceae species play key roles in the industrial production of vinegar [37]. The killing effect of tetracycline on Acetobacteraceae may maintain the pH of artificial feed, which plays a key role in the growth and development of certain bacteria [38]. Although the number of ASVs in the tetracycline treated *C. suppressalis* was greater than that in the CK group, the uniformity results showed that many ASVs contained very few tags (Additional file 1). These tetracycline specific ASVs were most likely bacteria that preferred alkaline or neutral environments, and their source was bacteria suspended in the air. Therefore, although they could be sequenced, only a few tags were available.

The high proportion of Enterococcaceae in the *C. suppressalis* suggests that they may play an important role in the host. The increase in the proportion of Enterococcaceae caused by antibiotic treatment (Fig. 1B) indicated that Enterococcaceae acquired multiple antibiotic resistance during long-term co-evolution in *C. suppressalis*. Enterococcaceae also dominate the microbial population in *C. suppressalis* and confer different pesticide tolerances [39], which may be related to the high pH value of the *C. suppressalis* intestine. The acetate secreted by Enterococcus reduces the pH of the *C. suppressalis* intestine and protects it from certain toxins [40]. For example, under alkaline conditions, the amino acid chains of the Bt protein crystals are specifically transformed into active peptide segments, thus enhancing the insecticidal activity of the Bt protein [41]. Plant-eating pests, especially lepidopterans, are mostly alkaline in the intestines, which is also an important reason why the target pests of Bt proteins are mainly lepidopterans. When the number of Enterococcus in the *C. suppressalis* was high, its tolerance to Bt proteins could be stronger. However, more research was needed to prove its resistance to other chemical pesticides, such as the chlorantraniliprole and methoxyfenozide used in this study. But, it can be inferred that the microorganisms that regulate the intestinal environment of *C. suppressalis* are necessary for pesticide tolerance.

The changes in symbiotic microorganisms caused by antibiotics did not affect the growth and development of *C. suppressalis* reared on artificial feed, which may be related to the easy availability of nutrients. *C. suppressalis* is an omnivorous species. In addition to rice, it can also harm water oats, corn, sorghum, millet, sugarcane, and other crops. *C. suppressalis* feeding on rice, water oat, corn and artificial feed has different symbiotic microorganisms [42], which may be related to the different growth, development, and fitness of the rice and water oat populations of the *C. suppressalis* [43, 44]. In some insects, the gut microbes determine the feeding habits of the host [45], particularly those that feed on cellulose. For example, termites, they can degrade lignocellulose using cellulose-degrading enzymes secreted by gut protozoa, symbiotic fungi, and bacteria, without which they cannot survive [46]. At the same time, the effects of antibiotics on the fitness of *Plutella xylostella* reared on artificial feed are significantly weaker than those reared on radish seedling [47]. Artificial feed is nutrient-rich and easily absorbed and does not require intestinal bacteria to facilitate digestion and degradation.

The symbiotic microbial changes caused by antibiotics are an important potential factor in the changes in the pesticide tolerance of *C. suppressalis*. This study demonstrates that the addition of antibiotics to artificial feed decreased the tolerance of *C. suppressalis* to chlorantraniliprole and methoxyfenozide (Fig. 4). There are two possible explanations for this decrease. The first is the direct toxic effect of antibiotics on *C. suppressalis* or the effect of similar pesticide synergists. Antibiotics mixed with pesticides were directly used to treat *C. suppressalis* that was fed without antibiotics, and the results showed that antibiotics had no direct effect on reducing the tolerance of *C. suppressalis* (Fig. 5). Therefore, this possibility is extremely low. The second is that the indirect effect of antibiotics on symbiotic microorganisms of *C. suppressalis* leads to changes in pesticide tolerance. A significant increase in the bioavailability of oral triazine herbicides in rats treated with ampicillin or cocktail therapy, and the gut microbiota altered by antibiotics, directly affect the increase in pesticide bioavailability by downregulating the expression of liver metabolic enzyme genes and upregulating intestinal absorption-related proteins [48]. Therefore, we conclude that the changes in symbiotic microorganisms caused by long-term antibiotic feeding are an important factor leading to changes in pesticide tolerance in *C. suppressalis.* If this symbiotic microbial change occurred in the wild population of *C. suppressalis* in the field, it may lead to a more significant decrease in pesticide tolerance compared to populations fed with artificial feed. As discussed earlier, microbiota plays a role in the dietary selection and growth and development of the *C. suppressalis* [38, 40], which may be closely related to plant secondary metabolites and nutrition. Sometimes, plant secondary metabolites acted as chemical pesticides on the *C. suppressalis* [49], and even some secondary metabolites are used as biopesticides, such as azadirachtin, matrine and osthole etc. Gut microbiota could help the herbivorous insects cope with these effects [50]. Therefore, changes in the gut microbiota could lead to a decrease in the ability of *C. suppressalis* to cope with adverse effects of secondary metabolism in rice. It was inferred that the gut microbiota of the wild population of *C. suppressalis* plays a more important role in its pesticide tolerance than artificially fed populations.

Symbiotic microbial changes caused by antibiotics are potentially important factors in the downregulation of detoxification metabolism-related genes in *C. suppressalis*. Insect symbiotic bacteria directly metabolize toxic substances or indirectly mediate the expression of host detoxification enzymes or related genes, thereby affecting the detoxification metabolism function of insects, enhancing their adaptability and competitiveness under environmental pressure and pesticide stress [51, 52]. For example, the gut symbiont *Citrobacter sp.* of *Bactrocera dorsalis* encodes phosphate hydroxylase and degrades the organophosphorus pesticide trichlorfon [53]. *Pseudomonas* in the *Hypothenemus hampei* enhances the resistance to caffeine, thereby increasing the harm to coffee fruits and reducing the impact of caffeine on the host [54]. In this study, we found that antibiotic feeding resulted in the differential expression of genes involved in the detoxification of chlorantraniliprole. This differential expression trend was not consistent in the CK and TET groups, but was significantly consistent in the CFM group. In the changes in symbiotic microbial diversity caused by antibiotic treatment (Fig. 2), the CFM group showed the lowest microbial diversity, especially the species richness index. The trends in the diversity of symbiotic microorganisms of *C. suppressalis* caused by antibiotic feeding were as follow: CK > TET > CFM. The trend of pesticide tolerance caused by different antibiotics was CK > TET > CFM, and the effect of different antibiotics on the gene expression of detoxification metabolism of *C. suppressalis* was CK ≈ TET > CFM. The consistency of the above three trends indicates that the change in symbiotic microbial diversity in *C. suppressalis* after antibiotic treatment is a potentially important reason for the decrease in pesticide tolerance and downregulation of detoxification metabolism genes of *C. suppressalis.*

## Conclusions

The role of symbiotic microorganisms in insects has always been a focus of research; however, studies on the role of symbiotic microorganisms in insect pesticide tolerance are still relatively few. Research on the symbiotic microbial community in *C. suppressalis* remains at the level of investigation into the infection status of geographical populations with different pesticide tolerance. In conclusion, this study identified the variation in symbiotic microbial diversity caused by different antibiotics and demonstrated that antibiotic treatment had no significant effect on the growth or development of *C. suppressalis* reared in artificial feed. At the same time, it was determined that the 72 h LC_50_ of the *C. suppressalis* was CK > TET > CFM, and direct treatment of the *C. suppressalis* in the CK group with antibiotics determined that the direct effect of antibiotics on the *C. suppressalis* did not lead to an increase in pesticide tolerance to chlorantraniliprole or methoxyfenozide. It was indirectly proven that the increase in pesticide tolerance of *C. suppressalis* reared for a long time was related to the changes in the symbiotic microbial community. RT-qPCR of potential genes related to chlorantraniliprole detoxification metabolism was performed, and it was determined that cefixime treatment reduced the expression levels of detoxification metabolism genes in *C. suppressalis.*, which is an important potential reason for the decrease in the pesticide tolerance of *C. suppressalis.*

### Electronic supplementary material

Below is the link to the electronic supplementary material.

Supplementary Material 1

## Data Availability

The raw reads of 16S rDNA amplicon sequencing have been deposited in the SRA database under BioProject accession number PRJNA1044162. The raw data are available in GenBank as Sequence Read Archive (SRA): SRR26926108 to SRR26926122.All other data generated or analyzed during this study are included in this published article.

## Abbreviations

TET: Tetracycline
CFM: Cefixime
16S rDNA: 16S Ribosomal DNA
LC_50_: Lethal Concentration 50
ASV: Amplicon Sequence Variant
